# Evaluating the safety, efficacy and complications of electrotherapy and its comparison with conventional method of hemorrhoidectomy 

**Published:** 2016

**Authors:** Payam Nikooiyan, Hamzeh Mohammadi Sardo, Bahram Poursaeidi, Motahareh Zaherara, Bijan Ahmadi

**Affiliations:** 1 *Department of Internal Medicine, Kerman University of Medical Sciences, Kerman, Iran.*; 2 *Department of Internal Medicine, Kerman University of Medical Sciences, Kerman, Iran.*; 3 *Specialty General Surgery, Department of Internal Medicine, Kerman University of Medical Sciences.*; 4 *Faculty of Medicine, Department of Anatomy, Bam University of Medical Sciences, Kerman, Iran.*; 5 *Specialty Gastroenterology, Department of Internal Medicine, Kerman University of Medical Sciences, Kerman, Iran. *

**Keywords:** Safety, Efficacy, Complication, Electrotherapy, Hemorrhoidectomy

## Abstract

**Aim:**

This study was performed to evaluate the efficacy, safety and complications of electrotherapy compared with conventional hemorrhoidectomy (Ferguson technique).

**Background:**

Ferguson hemorrhoidectomy is always associated with considerable pain and postoperative complications. Still, the electrotherapy method in which the hemorrhoidal tissue is not removed may not improve critical complications.

**Patients and methods:**

This randomized clinical trial was performed on patients with hemorrhoids referring to hospitals affiliated to the Kerman University of Medical Sciences during 2014-2015. One hundred and twenty patients presented with symptomatic hemorrhoids grade I, II, III, and IV were randomized into two groups. Group 1 (60 patients) underwent electrotherapy using 30 mA direct current and group 2 (60 patients) were submitted to Ferguson hemorrhoidectomy. The groups were compared regarding postoperative pain severity and complications, including recurrent symptoms, infection and recovery time to return to normal activities. The p≤ 0.05 was considered statistically significant.

**Results:**

More than 70% of patients in group 2 complained of severe pain, but in group 1, no more than 30% of patients experienced severe pain up to 6 hours post-surgery and 70% complained of mild pain 2-3 days post-surgery. Twenty four-hour hospitalization in group 2 and group 1 were 97% and 78%, respectively, whilst patients in electrotherapy group could be treated as outpatients. The mean return time to usual activities was 15 and 1.5 days for group 2 and 1, respectively.

**Conclusion:**

Electrotherapy with a direct current of 30 mA significantly reduce postoperative pain and the recovery period. This method showed a good success rate and less complication than the Ferguson method. As a result, because of more effectiveness, less pain, as well as shorter recovery time and getting back to normal activities, we recommend this procedure for the treatment of symptomatic hemorrhoids grade I, II, and III.

## Introduction

 Hemorrhoids are cushions of submucosal tissue containing venules, arterioles, and smooth muscle fiber. It seems that these submucosal cushions play a critical role in continence mechanism, causing complete closure of the anal canal at rest. Hemorrhoidal disease is one of the most common diseases of the anal region and constitutes about 50% of colorectal clinic visits. It can be occurred at any age and in both genders equally. There has been an evidence of physiologic changes of the anorectal with the development of hemorrhoids (1-5). This is followed by excessive straining, hard stool and increased abdominal pressure leading to congestion and prolapse of hemorrhoidal tissue (6). These patients usually complain of full rectum contraction feeling, mucus discharge, and bright red rectal bleeding (7). Internal hemorrhoids are located proximal to the dentate line and are graded based on the severity of the prolapse.

 In grade 1: hemorrhoids bulge into the anal canal, grade 2: hemorrhoids prolapse through the anus, but reduce spontaneously, grade 3: hemorrhoids prolapse through the anal canal and require manual replacement, and grade 4: irreducible hemorrhoids that are permanently prolapsed (8). Several methods have been developed for the treatment of symptomatic hemorrhoids, including diet changes and surgical and non-surgical techniques (9, 10). Approximately, 70% of patients undergo conventional hemorrhoidectomy as the most definitive treatment of this disease. Pain and wound healing process are two common post- surgery side effects (11), which cause delay in discharge, more post-surgical medication and frequent hospital admissions (12), and discomfort for the patient that lead to the patient's physical and mental disturbance (13). Reducing the factors that are associated with pain and healing process and lead to economic, social and psychological consequences can decrease potential harms. Consequently, multiple non-surgical methods that potentially alter physiological characteristics (14-18) have been developed, including tissue fixation techniques (sclerotherapy, cryotherapy, and photocoagulation), as well as rubber band ligation and electrotherapy.

But the multiplicity of treatment options represents the fact that none of these methods results in an effective treatment and despite their role in controlling symptoms, recurrence is their common defect. Also, little information is available about possible physiological and clinical abnormalities in patients with grade I and II hemorrhoids, who undergoes direct electrotherapy (20-26).

This study aimed to investigate the complications and effectiveness of two surgical methods in patients with complaints of the mucosal protrusion, blood dripping, and a sensation of incomplete evacuation referring to the endoscopy unit of Afzalipour hospital and Besat clinic in Kerman and after the diagnosis of hemorrhoids were referred to the surgery department for subsequent therapeutic interventions.

Also, given the importance of the incidence of hemorrhoids in Kerman and its cultural and economic aspects, this study aimed to investigate the effectiveness, safety and complications of electrotherapy in comparison to conventional hemorrhoidectomy (Ferguson method) to improve decision-making and planning a better health care in the city of Kerman and highlighting at the national and international levels. 

## Material and Methods

In this randomized clinical trial (the Ethical code IR.kmu.REC.1394.27), patients with hemorrhoids referred to hospitals affiliated to Kerman University of Medical Sciences during the years 2014-2015 were enrolled. Patients underwent complete physical and digital rectal examination and anoscopy and/or rectosigmoidoscopy after obtaining informed consent.

Using a randomized block design, patients were allocated to the two treatment groups (n=60) of electrotherapy and conventional hemorrhoidectomy (Ferguson method).

Those with signs of the fissure, anal stenosis, fistulae, abscesses, polyps, cancer and previous surgery on anal area were excluded. The patients in the first group were treated using electrotherapy. With the patient in the lithotomy position, hemorrhoid cushions were specified by anoscopic examination. Then, hemorrhoidal cushions were grasped with forceps and by using the movement of electrons with a direct current voltage of 30mA between the probes; we attempted to destroy hemorrhoids (not recommended in the case of grade IV hemorrhoids). 

All patients were visited on days 1, 7 and 21 after the procedure and pain intensity (according to visual analog scale rating from 0 to 10) (27)), wound healing (restoration), and patient satisfaction were assessed. Treatment completion was when the symptoms were resolved and patient/ physician satisfaction to be met (successful treatment), or due to failing to achieve the above objectives, failure to be regarded. If re-treatment was needed, after a period of successful treatment, recurrence was considered. The patients in the second group were treated with conventional hemorrhoidectomy. In this group, patients received anesthesia in a lithotomy position and a maximum of two hemorrhoidal cushions were surgically removed. Possible complications after the treatment in both groups of patients were explained. After the treatment period, symptoms including protrusion, pain, and bleeding after bowel movement, complications (pain, bleeding, and anal stenosis), recurrence, failure (non-response treatment) or the success of treatment (removal of symptoms) were examined. Finally, data were analyzed using SPSS v.16 statistical software and statistical tests, including t-test, Fisher's exact test and Chi-Square. P < 0.05 was considered as statistically significant. 

## Results

In this study, 120 patients were studied, of which 60 were operated by electrotherapy (group 1) and 60 with hemorrhoidectomy (Ferguson) (Group 2). A total of 79 patients (65.8%) were male and 41 (34.2%) were female and the male to female ratio was 1/1.9. Patients aged 30 to 68 years and the mean age was 40.9. Of these patients, 26 patients had a history of heart disease, which was relatively similar in two groups (8 cases of treated blood pressure, 2 cases of heart disease, 4 cases of managed diabetes, 3 cases of kidney stones, 4 cases of chronic anemia; hemoglobin less than ten, 3 cases of inguinal hernia surgery, and 2 cases of appendectomy). Two of the patients were taking aspirin due to underlying disorder which was discontinued from seven days before up to one week after the treatment. One of the patients had a positive family history of gastric cancer. A total of 26 patients, according to the medical history, physical examination, and potential cases underwent colonoscopy, and no abnormal findings were reported. The most common symptoms were bleeding (77.5%), pruritus (45.8%), discharge (41.6%) and protrusion (37.5%), and the lowest was anal pain (22.5%) respectively (table 1). Patients often had multiple symptoms, but the most common cause of referral was bleeding (table 2). All the patients suffered from symptomatic internal hemorrhoids and no statistically significant difference was observed between patients for primary signs of disease, bowel movement habits, diet, and the number of hemorrhoidal cushions. In group 1, all patients were treated with electrotherapy without any acute problem, of them 18 cases underwent spinal anesthesia and 42 cases underwent general anesthesia (table 3). None of the patients in this group were hospitalized for more than twelve hours of operation. 

In this group 10 patients had one cushion of symptomatic hemorrhoid, 24 patients had two cushions of symptomatic hemorrhoid, and 26 patients had three cushions of symptomatic hemorrhoid. Totally, 58 patients were treated in one session, and 2 patients in two meetings with an average interval of 1 week. In the group 2, 12 patients had one cushion of symptomatic hemorrhoid, 29 patients had 2 and 19 patients had 3 cushions of symptomatic hemorrhoid that all of them were operated in one session except one. In this group, 48 patients were operated with local anesthesia and 12 patients underwent surgery under the general anesthesia. All patients were discharged from the hospital 24 hours post-surgery. In this group, one patient was hospitalized again and underwent surgery and hemostasis three days after discharge, due to bleeding and severe prolapse. Regarding the complications the day after surgery, the pain was the most significant complication in the group 1. Bleeding and constipation ranked the next. In group 2, the most common complication was postoperative pain and bleeding and discharge were the next (table 4). However, bleeding, discharge, and constipation were significantly higher in the Ferguson group than the electrotherapy group (table 2). Pain severity was significantly greater in group 2 (P <0.0001). Post-operative fever was not seen in the first group. However, temperature above 38.3 °C was reported in 5 patients in the second group; not accompanied by wound infection. Still, bleeding, discharge and constipation were significantly more frequent in the group 2. At the end of the first week of operation, pain intensity, the need for analgesics, the amount of bleeding, and anal secretion were significantly lower in the electrotherapy group. However, in the Ferguson group, prolapsed hemorrhoids occurred to a lesser extent (Table 3).

**Table 1:** Specification of demography and Presenting symptoms

* Data were shown mean ±sd,

† based on independent test

**Table 2:** First-day post operation follow up (complication

Post operation symptom*	Electrotherapy [n(%)]	Ferguson [n(%)]	P value†
Fever	0	5 patients (8.3)	0.057
Discharge	0	16(26.6)	<0.001
Incontinence (gas(	0	3(5)	0.244
Pain	60(100)	60(100)	…
Constipation	4(6.6)	15(25)	0.011
Bleeding	4(6.6)	18(30)	0.002
Urinary retention	0	2(3.3)	0.496
prolaps	12(20)	8(13.3)	.195
Pain score	1.5±0.41	6.23±1.30	<0.001

* Data were shown frequency (percent),

† based on chi-square test

**Table 3.** First-week post operation follows up (complications

Improvement*	Electrotherapy [n(%)]	Ferguson [n(%)]	P value†
Fever	0	0	
Discharge	8(13.3)	35(58.3)	<0.001
Incontinence (gas(	0	2(3.3)	.496
Pain	6(10)	37(61.6)	<0.001
Bleeding	4(6.6)	10(16.6)	0.153
Urinary retention	0	0	
Pain score	0±0	2.7±0.44	<0.001
Prolapse	41(68.3)	2(3.3)	<0.001

* Data were shown frequency (percent),

† based on chi-square test

**3rd week post operation**

**Table 4:** 3^rd^ week post operation follows up (complications

Improvement *	Electrotherapy [n(%)]	Ferguson [n(%)]		P value†
Fever	0	0		…
Discharge	6(10)	0		0.027
Incontinence (gas)	0	2(3.3)		0.496
Pain	0	23(38.3)		<0.001
Constipation	10(16.6)	11(18.3)		0.810
Bleeding	1(1.6)	8(13.3)		0.032
Urinary retention	0	0		...
Pain score	0±0	0.53±0.09	<0.001	
Prolapse	0	2(3.3)	0.496	
Off days	1.5±0	15.53±7.84	<0.001	

* Data were shown frequency (percent),

† based on chi-square test

**Table 5:** 6^th^ week post operation follow up

	Electrotherapy [n(%)]	Ferguson [n(%)]	P value†
Pain	0	0	0.119
Prolapse	0	0	…
Discharge	0	0	…
Bleeding	1(1.6)	4(6.6)	0.364
Patients without complications	59((98.3%)	55(91.6%)	0.324
Recurrence of symptoms	1(1.6%)	5(8.3%)	0207

* Data were shown frequency (percent),

† based on chi-square test

At the end of the third week, none of the patients complained of pain, fever, protrusions, discharge, incontinence (gas), and urinary retention and ten cases of constipation and one case of bleeding and recurrence (grade 4) were reported. In the second group, there was no sign of fever and infection. Yet, 23 patients complained of pain, 11 of constipation, 8 of occasional bleeding, 6 of occasional discharge and 2 of incontinence (gas). Regarding pain, discharge and bleeding, the difference was statistically significant. There were no significant differences between the two groups regarding keeping control of bowel movements, pre and post- operation, in three-weeks of the follow-up period. It took 1.5 day for patients in group 1 and 15.5 days for patients in group 2 for getting back to normal daily activities that the difference was statistically significant (p< 0.05, table 4). After five to six weeks of follow-up, there was no statistically significant difference in terms of pain, protrusion, discharge, and bleeding between the two groups (Table 5). Overall, the difference between the amount of pain, bleeding, anal hygiene, and protrusions after the surgery at the end of the first week was significant in the two groups; but it was not significant at the end of the third week. 

## Discussion

Electrotherapy method with a direct current of 30 mA can considerably reduce the postoperative pain and the time taken to return to normal activities. It had a good success rate and fewer complications compared to the Ferguson method and can be used to treat symptomatic hemorrhoids grade I, II, and III.

Various methods have been proposed to be effective for treatment of hemorrhoids. This variation in therapeutic procedures suggests that none of them are completely successful. Different studies have compared these approaches and have presented different results (28-30). Hemorrhoidectomy is known as the most decisive option, particularly in the treatment of advanced cases of hemorrhoids (grade three and four). However, this method compared with other methods is associated with significant pain and high rates of complications and leads to severe changes in the anal natural physiology (9, 29, and 31). Currently, this method is the procedure of choice for the treatment of patients who do not respond to other clinical practices, do not tolerate these treatments, or experience grade III and IV hemorrhoids or external skin flap at the same time (32). Hemorrhoid is a completely benign condition. Yet, the treatment should offer the minimal invasion and the highest degree of safety and recovery time for the patient. Accordingly, we used electrotherapy in the treatment of symptomatic patients with internal hemorrhoids grade I, II, III, and IV. When selecting the most appropriate treatment options, the successful results of this method should be weighed against potential side effects. A method can be successfully measured by exploring the recurrence rate, complications after surgery and getting back to a normal activity. Several retrospective and controlled clinical trials have been carried out from 2004 to 2010 to compare the operation period, postoperative pain, urinary retention and returning to normal activities in two methods of electrotherapy and conventional hemorrhoidectom. Electrotherapy has been reported more preferable than the conventional surgery method. Izadpanah, *et al.* conducted three studies in Shiraz to evaluate electrotherapy treatment. In their first study in 2004, they concluded that postoperative pain was mild and tolerable and 93.2% of patients returned to normal activities after two days of surgery, and electrotherapy was introduced as a safe and effective method without any major complications in these patients. This method was used for the treatment of hemorrhoids grades I and II (24). One year later, a randomized prospective study was conducted to compare electrotherapy with the Ferguson method and it was discovered that the method of electrotherapy using a direct current of 30 mA can significantly reduce the pain and the duration of surgery and hospitalization. This procedure had a high success rate and less complication compared with the Ferguson hemorrhoidectomy resulting in security and prosperity (23). In the other study in 2010 for comparison of electrotherapy, rubber band ligation and hemorrhoidectomy in a clinical and manometric study they concluded that electrotherapy was a safe, effective and simple treatment for grades I I and III internal hemorrhoids that reduced postoperative pain and complications, and had a minimum changes in anorectal manometric features compared with other methods(31). The results of all these studies correspond with the present results. These findings of the current study are consistent with those of Olatoke, et al. who found that direct current electrotherapy was an effective and painless treatment method for grades 1 to 3 internal and mixed hemorrhoid disease (32). In a study by Kandilarov and Dimitrova, it has been concluded that there are different treatment procedures, including surgical, for the treatment of the hemorrhoidal disease. However, the selection of the therapeutic method, establishing the best-individualized therapy, depends on the surgeon’s decision (33). Contrary to our results Majeed, et al. found that regarding wound healing recurrence time and post-operative complications, there was no significant difference between open and closed haemorrhoidectomy (34). In a narrative review, Picchio, et al. concluded that in any surgery, further care and more advanced facilities, as well as more accurate experiments, would increase the efficacy and safety of the procedure. Higuero, et al. (2016) proposed that in the treatment of hemorrhoids, fiber diet should always be in the first intention and instrumental treatment should be performed only if medical treatment fails (except in grade ≥III prolapse) and surgery should be the last resort, and the patient should be well informed of the surgical alternatives, including the possibility of elective noninvasive methods. 

 Studies on the prevalence of hemorrhoid disease, emphasizes that this disease occurs equally in both genders (20, 37, 39). In the present study, 65.8% of the patients were male and 34.2% were female. Although gender differences corresponded with some studies (40-42), but it cannot be generalized to the whole population due to the small sample size. In this study, patients were included those with symptomatic internal hemorrhoid grade one, two, three and four, and symptoms included bleeding, discharge, constipation, pain, and itching, respectively. These results were consistent with previous studies (23,31). Also, in this study, the effectiveness of both treatments has been confirmed. At the end of the three weeks follow-up, a total of 95% of patients were completely treated and no significant difference was observed between the two groups. In the group treated by electrotherapy, the treatment deemed successful in 98.3% of patients. Other researchers that used different degrees of electrotherapy have reported a complete treatment in 97.1% of patients. The result of our study is comparable with these results. The most important difference between the two groups was in the postoperative pain. At the end of the first week of treatment, there was a significant difference between the two groups in terms of pain incidence, the severity of pain, and analgesic consumption. Frequency and severity of bleeding in the group 1 were less than the group 2, and urinary retention was seen only in patients treated with hemorrhoidectomy. The cushions prolapse in the first group at the end of the first week was more than the second group. This is due to thrombosis of hemorrhoidal  veins caused by electrotherapy. This condition was less seen in the Ferguson method. Most prolapsed hemorrhoids shrank back and became small at the end of the third week. The life-threatening complication was not observed in the group treated by electrotherapy. These results have also been obtained in previous studies (23, 31). Also, in this study, there was no postoperative infection, probably due to the use of antibiotics in all patients. Given that hemorrhoidectomy was performed using anesthesia, complications of anesthesia in this group of patients was not surprising that has not been investigated in this study. Conflicting reports on the prevalence of constipation in postoperative follow-up might be due to confounding factors such as diet, addiction, and mobility of patients. Returning to work is an important variable in the health of individuals. However, due to a variety of social and economic factors, it will be an unreliable variable in the evaluation of treatment success. Therefore, in this study for a closer look, the patient returning to normal activities, representing the recovery time of patients was studied. In this study, patients who were treated with electrotherapy were able to resume normal activities almost after a daybreak. However, in the other group who were treated with hemorrhoidectomy patients returned to normal activity for an average of 15.5 days after the treatment. The quality of life is affected by many factors, including the demographic factors, socioeconomic status, culture, jobs and people's expectations. In this study, due to the inability to find a reliable scale to measure the quality of life-based on the regional, social and cultural condition, this variable was excluded. In this study, patients were evaluated in a five to six week period and by the end of this period and at the end of this period, 59 patients (89.3%) in group I and 55 patients (91.6%) in the second group had complete recovery. Overall, six cases of symptomatic cases were reported, one in group I and 5 in group, II. In a study conducted by Izadpanah,* et al.* (2004-2005) in the 2015 surveys of electrotherapy carried out in Shiraz, 1.3%(27 cases) of recurrence was reported in two weeks up to two months postoperative. Only 0.1 %( 3 cases) respond t not adequately and underwent hemorrhoidectomy. Izadpanah, *et al.* have shown that patients with recurrence of symptoms treated by electrotherapy may be treated again with the same treatment (23, 31). In the present study, four cases of recurrence were found by the end of the first week in electrotherapy group that was more associated with bleeding. All four cases had developed grade IV hemorrhoids, which were re-subjected to electrotherapy and at the end of the third week, only one patient did not respond to treatment and was scheduled for hemorrhoidectomy. Although these results were not statistically significant, but it can be concluded that grade IV hemorrhoid may best be treated with the surgical procedure; given the high risk of recurrence rate. Although the study was conducted on a small sample size and short follow-up period, results showed high satisfaction by patients in electrotherapy group. Also, comparing the two treatment groups at the end of the follow-up period indicates similar results and effectiveness of the two methods. Therefore, it can be concluded that the use of electrotherapy, due to fewer complications and earlier recovery and return to normal activity can be considered as the first line of treatment in patients with symptomatic internal hemorrhoids grade I, II, and III.
